# Metabolic reprogramming and epigenetic effects due to reducing sugars and glycation products in cancer

**DOI:** 10.3389/freae.2025.1752493

**Published:** 2026-01-27

**Authors:** Savita Bansal, Archana Burman, Taruna Arora, Meenakshi Vachher, Nalini Moza Wali, Bhupender Kumar

**Affiliations:** 1 Department of Biochemistry, Institute of Home Economics, University of Delhi, New Delhi, India; 2 Department of Microbiology, Swami Shraddhanand College, University of Delhi, New Delhi, India

**Keywords:** AGE-RAGE axis, cancer metabolism, epigenetics, glycation, metabolic reprogramming, reducing sugars, oxidative stress

## Abstract

Cancer cells have the ability to reprogram their metabolism to meet the proliferative and survival demands. These metabolic alterations involve the formation of many active metabolites associated with epigenetic modifications or remodelling together driving tumourigenesis through self-perpetuating feedback loops. Among the various metabolic stressors, reducing sugars, principally glucose and fructose, lactate, acetyl-CoA, glycolytic intermediates such as 3-deoxyglucosone, glyoxal, polyol pathway metabolites, and methylglyoxal effectively modulate the chromatin remodelling and gene expression. These processes lead to dysregulated DNA methylation and histone modifications involving acetylation, methylation, polysialylation, lactylation, and glycation establishing a tumourigenic environment. Elevated levels of reducing sugars and glycolytic intermediates also contribute to the formation of a large group of reactive molecules termed advanced glycation intermediates (AGIs) and advanced glycation end products (AGEs), which interact with their receptor RAGE to activate signalling cascades resulting in oxidative stress, inflammation, and aberrant gene regulation. Furthermore, the AGE-RAGE axis reprograms cancer metabolism influencing key signalling pathways including PI3K/AKT/mTOR and NF-κB. The epigenetic alterations and metabolic perturbations induced by reducing sugars and non-enzymatic glycation reactions also influence the tumour microenvironment (TME) through extracellular matrix (ECM) remodeling, angiogenesis, and immune evasion. This review elucidates the crosstalk between metabolic reprogramming, AGE-RAGE-mediated signalling, and epigenetic modulation that forms a complex network associated with cancer initiation, progression, and resistance to therapy. Understanding the molecular interplay between these pathways could pave the way for novel metabolic and epigenetic therapeutic strategies aimed at disrupting this vicious cycle and impeding tumour growth.

## 1 Introduction

In normal cells, different cellular processes are under fine regulation, and certain dysregulation could turn a normal cell into a neoplastic cell. Metabolic dysregulation is one of the hallmarks of cancer cells (Hanahan, 2022). Rapidly growing cancer cells require a large quantity of cellular building blocks and energy to sustain their growth which they achieve through metabolic reprogramming. The intermediates of glycolysis being central to metabolism serve as the precursors to these building blocks (Gatenby and Gillies, 2004). Swift utilisation of glucose and consequent lactate production even in the presence of oxygen via the Warburg effect is a hallmark of cancer cell metabolism (Warburg et al., 1927). This rapid utilisation of glucose provides quick ATP via substrate level phosphorylation to support the need for rapid cancer cell division. Along with glucose, fructose is also known to promote anaerobic glycolysis thereby favouring cancer cell proliferation (Nakagawa et al., 2020). Cancer cells generally have an increased activity and expression of many enzymes including hexokinase 2 (HK2), pyruvate kinase M2 (PKM2) and lactate dehydrogenase A (LDH-A) isoenzyme which together ensure a high rate of glycolysis and availability of its intermediates (Hay, 2016). These enzymes and associated metabolites are also known to alter the epigenetic state of DNA and chromatin in cancer cells (Miranda-Goncalves et al., 2018; Verma and Lindroth, 2025; Wang P. et al., 2024).

Epigenetic signature of DNA and chromatin in eukaryotes is dynamic in nature wherein the local and global signature is known to alter in conditions such as cell division, metabolic disorders, obesity and cancer (Eden et al., 2003; Feinberg and Vogelstein, 1983; Jones and Baylin, 2002; Kaimala et al., 2025). Such alterations in the DNA/chromatin drastically affect the metabolic behaviour of cells. Apart from merely feeding the tumours, high glucose and fructose alter the level of several cofactors including acetyl-CoA, UDP-GlcNAc, α-KG, NAD^+^, SAM, and lactate to modulate the chromatin structure (Verma and Lindroth, 2025). These cofactors in turn affect the DNA and histone modifications such as DNA methylation, histone methylation, acetylation, phosphorylation, which subsequently alters cancer cell metabolism and promotes cancer proliferation (Wu et al., 2018). Thus, there exists a dynamic relationship between epigenetic state and metabolism (Haws et al., 2020).

Apart from altering the cofactor level, reducing sugars also produce other reactive intermediates that have the potential to affect the epigenome (Perrone et al., 2020). Reducing sugars such as glucose and fructose and their reactive metabolites are known to produce AGEs during metabolic dysregulation in cancer which in turn are recognized to promote cancer aggression and progression (Shen et al., 2024). Glucose and fructose both can produce AGEs though fructose is chemically more reactive than glucose in producing AGEs (Gugliucci, 2017). AGEs show their detrimental effects by interacting with receptors, namely, receptor of advanced glycation end products (RAGE) causing a pro-inflammatory signalling cascade. Several studies have noted a higher predisposition towards metastasis and poor prognosis in cancer patients having diabetes compared to non-diabetic cancer patients (Shahid et al., 2021). Long term hyperglycaemia and corresponding increased AGEs suggests a prevailing connection of AGEs with increased metastatic potential of cancer cells (Rojas et al., 2018). The present review focuses on altered metabolic pathways in cancer cells, highlighting how a sugar-rich environment (glucose/fructose) and glycolytic intermediates contribute to the accumulation of AGEs, which in turn induce oxidative stress, inflammation, and extracellular matrix remodelling. This review also delineates the AGE-RAGE-mediated pathways that drive the oncogenic environment inside the cells budding from altered rates of metabolic pathways, glycolytic stress and epigenetic effects. These changes synergistically support the cancer metabolism promoting tumour growth, survival, and progression (Figure 1).

**FIGURE 1:**
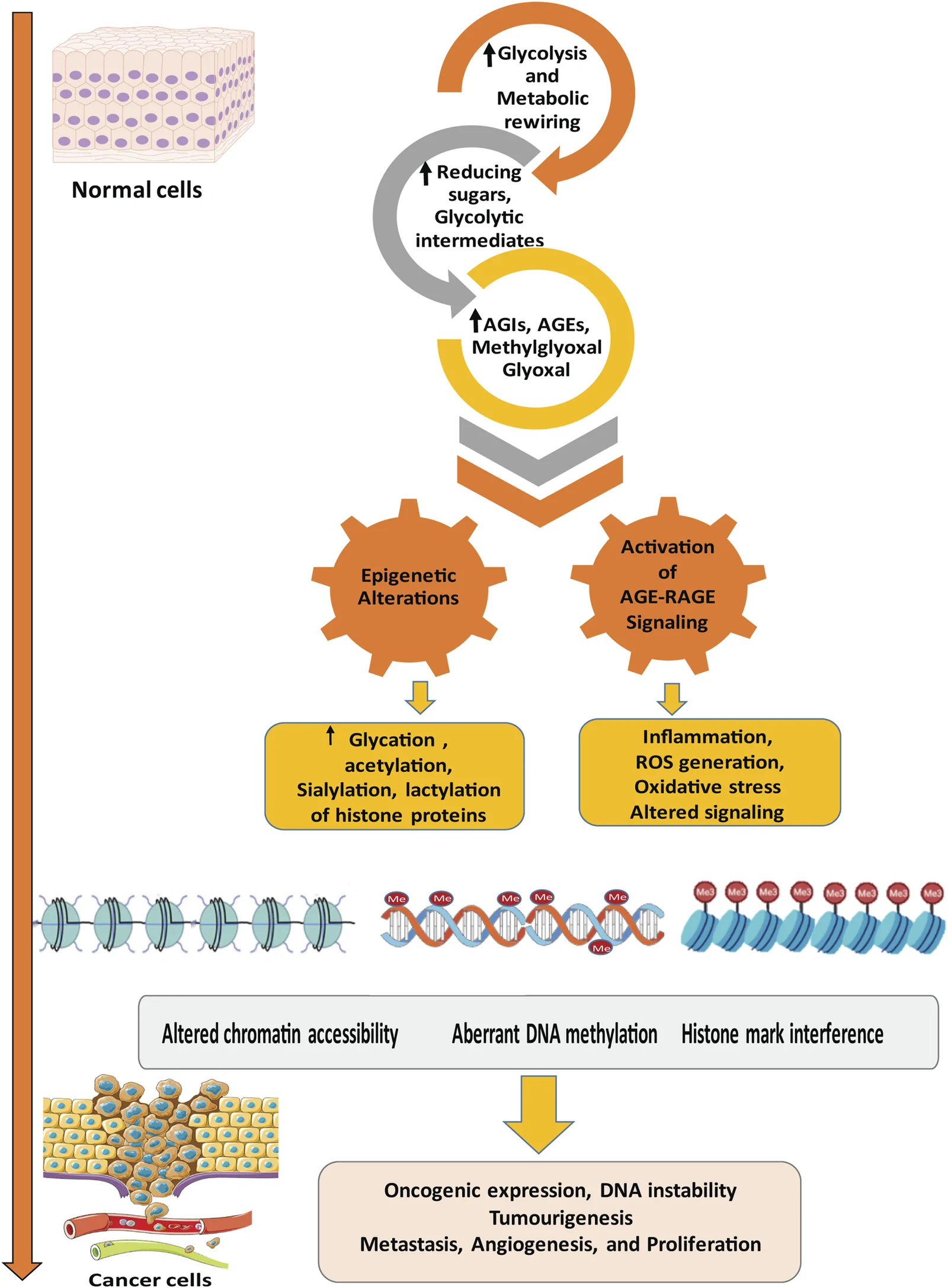
Schematic representation of the cascade associated with altered metabolic rewiring, reducing sugars utilisation, advanced glycation products formation and mediated epigenetic changes. Elevated glycolysis leads to the accumulation of AGIs like methylglyoxal and glyoxal. These AGIs are capable of glycating histones (H3, H4), methylation enzymes (DNMTs, HATs) and DNA bases which results in changed chromatin accessibility, histone code interference and abnormal DNA methylation. Simultaneous up regulation of AGE-RAGE signalling leads to activation of various altered signalling cascades, inflammation and generation of reactive oxygen species (ROS). The synergistic effects of these lead to facilitation of oncogenic environment exhibiting DNA instability, angiogenesis, invasion and increased metastatic potential.

## 2 Reducing sugars, epigenetic and cancer

### 2.1 Elevated glucose concentration, cancer metabolism and epigenetics

Glucose serves as the primary energy source for normal cells and as discussed above even more so for cancer cells. Metabolism of normal cells is regulated through hormones like insulin and insulin-like growth factor-I and II (IGF-I and IGF-II) (Gallagher and LeRoith, 2010). Certain metabolic disorders like type II diabetes (T2D), metabolic dysfunction associated fatty liver diseases (MAFLD) and obesity are known to promote tumourigenesis due to altered metabolism and associated epigenetic changes (Cheng et al., 2018; Long et al., 2024; Moslehi and Hamidi-Zad, 2018). Some cells depend on insulin signal for internalisation of glucose whereas brain, liver, RBCs, kidney, endothelium are independent of insulin and hence more vulnerable to hyperglycaemia associated complications (Mallik et al., 2024). PPAR γ (known to regulate metabolism) and P16 (known to regulate cell cycle) promoter hypermethylation driven by hyperglycaemia is reported to promote colorectal and pancreatic cancers (Porcuna et al., 2021; Sun et al., 2023; Xing et al., 2013). Hyperglycaemia is known to promote histone O-GlcNAcylation which favours oncogenic gene expression via altered chromatin accessibility (Chomphoo et al., 2023). Hyperglycaemia is thought to mediates its pro-cancerous epigenetic effect through disrupting the activity of two key enzymes, adenosine monophosphate (AMP)-activated protein kinase (AMPK) and tet methylcytosine dioxygenase 2 (TET2) (Wu et al., 2018).

In normal cells AMPK is known to act as glucose and energy sensor while TET2 is the main DNA demethylating enzyme (Hardie et al., 2012; Wang et al., 2018). TET2 is an α-ketoglutarate- and Fe^2+-^dependent dioxygenase (α-KGDDs) epigenetic enzyme maintaining DNA methylation by converting 5-methylcytosines (5mCs) to 5 hydroxymethylcytosine (5hmC)(Rasmussen and Helin, 2016). Under physiological conditions AMPK mediates phosphorylation of TET2 thereby protecting it and maintaining its tumour suppressive activity. But in cancers like hematological malignancies, particularly myeloid neoplasms, high glucose levels cause AMPK inactivation leading to destabilization of TET2 causing hypermethylation of DNA (Wu et al., 2018). The intrinsic TET2 mutations in mouse and human studies were demonstrated to be activated through extrinsic factors like hyperglycemic stress as seen in acute myeloid leukemia (AML) and Myelodysplastic Syndromes (MDS) wherein the TET2 destabilization leads to further aggravation of preleukemic status (Arcidiacono et al., 2012; Cai et al., 2021).

The ability of biological system to modify the cellular metabolism in response to nutrient availability is central to sustaining metabolic homeostasis. Glucose, fatty acids, acetate, glutamine and lactate can contribute to acetyl-CoA production. It is a central metabolite and the primary acetyl group donor for histone acetylation influencing histone acetylation and genomic functions impacting cancer progression (Feron, 2019; Shi and Tu, 2015). Overproduction of acetyl-CoA causes the histone acetyltransferases (HATs) to promote global histone acetylation which modifies the chromatin structure, making it easily accessible to transcription factors thus causing hyperexpression of growth-related genes ultimately promoting tumourigenesis (Lee et al., 2013; Miziak et al., 2024). Excess glycolysis in cancerous cells leads to lactate accumulation, which is responsible for the acidification of the tumour cell microenvironment. Lactate is also involved in suppressing anti-tumour immune response, supporting drug resistance, invasion and inflammation, along with epigenetic modifications like lactylation (Chen et al., 2024).

Glucose is the main fuel source in cancer cells and its role in epigenetically mediated tumour cell regulation requires further investigation. Silent Information Regulator 1 (SIRT1) a NAD^+^-dependent class III histone deacetylase enzyme maintains glucose homeostasis, regulates deacetylation and act as key player in the progression of malignant tumours (Hashemi et al., 2025). SIRT1 suppresses tumours and its lowered expression is associated with poor prognosis in patients with glioma (Chen et al., 2019). Recently, it was reported that high concentrations of glucose in cancerous cells can promote the proliferation, migration, and invasion of glioma cells, by down-regulating the SIRT1 expression leading to increased levels of acetylated High Mobility Group Box 1 (*HMGB1)*(Wang Y. et al., 2024). On the other hand, deacetylation of the histones has also been reported in diabetes and cancer through the altered NAD^+^/SIRT1 pathway (Kitada and Koya, 2013; Liu and McCall, 2013). The availability of NAD^+^, the cell’s energy equivalent, is critical for SIRT1’s activity (Canto and Auwerx, 2012). SIRT1 removes acetyl groups from histone proteins especially H3K9 and H4K16, involved in gene expression and regulation. The deacetylation of these histones creates a more condensed chromatin structure, thus inhibiting gene transcription. This enzyme also deacetylates nonhistone proteins like p53, FOXO typically implicated in cancer and diabetes (Lee and Gu, 2013; Voelter-Mahlknecht and Mahlknecht, 2010).

SIRT1 is known to play roles of both tumour promoter and suppressor based on cellular context and type of cancer (Deng, 2009). SIRT1 expression is found elevated in certain breast and colorectal cancer subtypes where it promotes cancerous growth by deacetylation and inhibiting p53-like tumour suppressor proteins (Dong et al., 2024; Rifai et al., 2017). Cancer spreads as a result of the inhibition of apoptosis and regulatory pathways linked to metastasis and drug resistance. In other cancers like gastric, hepatocellular carcinoma (HCC) and bladder carcinoma, and some cases of breast and ovarian cancers, based on unique cellular locations, SIRT1 expression is downregulated (Farcas et al., 2019; Hu et al., 2017; Yang et al., 2013). In gastric cancers the growth is inhibited through downregulation of the oncogene ARHGAP5 expression and the transcription of Cyclin D1 (CCND1) via NF-κB pathway (Yang et al., 2013). In HCC based on the cellular localization of the upstream and downstream molecules, SIRT1 reflects dual behaviour which needs further investigation (Fang and Nicholl, 2011).

High intracellular glucose also activates the polyol pathway (PP) that diverts glucose metabolism toward fructose production, promoting oxidative stress, inflammation, and chemoresistance. Schwab et al. (2018) reported a connection between glucose-transforming PP and epithelial-to-mesenchymal transition (EMT) in cancer cells which enables them a strongly invasive and drug-resistant phenotype (Schwab et al., 2018). Polyol pathway involves the conversion of glucose into fructose by two step enzymatic reactions catalysed by aldose reductase (AKR1B1) and sorbitol dehydrogenase (SORD) (Hers, 1956). Under normoglycemic conditions the activity of PP is seems to be negligible but under hyperglycemic conditions, such as diabetes its activity is increased and is associated with numerous diabetic complications (Gonzalez et al., 1984; Morrison et al., 1970). Due to the glucose dependency in cancer, as well as the role of oxidative stress-induced inflammation, PP can be regarded as an excellent target to study the progression of cancer. Importance of PP in tumourigenesis has also been demonstrated in various studies where the impairment in the function of the rate-limiting enzyme of PP, AKR1B1 diminishes cell growth, formation of precancerous lesions, migratory capabilities as well as metastasis (Tammali et al., 2006; Tammali et al., 2011). Recently, Schwab et al. (2025) also explored the importance of PP in genetic and pharmacological manipulations studies in *in-vitro* and in murine xenograft models where they revealed that PP activity is indispensable for non-small cell lung cancer (NSCLC) growth and survival (Schwab et al., 2025). PP deficiency was found to be responsible for multifactorial deficits associated with ATP deficiency and DNA damage leading to induction of apoptosis. They also showed that fructose produced by the PP as well as other non-glycolytic hexoses, promotes cancer cell survival and resistance to chemotherapy by maintaining NF-κB activity and inducing an oxidative metabolic shift (Schwab et al., 2025). Considering the importance of PP on cancer cell growth and survival, it can be considered as a candidate for future therapeutic approaches against cancer progression.

High glucose concentration and reactive glycolytic intermediates are also associated with increased production of AGEs. These are the complex group of compounds that can be formed through different mechanisms involving Maillard reaction, glucose oxidation and peroxidation reactions and through the polyol pathway (Bansal et al., 2023). The importance of AGEs in development and progression of cancer has drawn the attention of researchers in recent years. AGEs can mediate several epigenetic effects in cancer cells via different mechanisms such as upregulation of the expression of TET1 which is involved in DNA demethylation and the induction of stress and activation of downstream signalling pathways which are further discussed in the later sections (Turner, 2017; Wu et al., 2023).

#### 2.1.1 Lactylation and epigenetics

Altered metabolism in cancer cells also entails increased production of lactate which promotes lactylation, wherein lactate molecules bond to lysine residues on histones and other proteins. This post translation modification (PTM) is noteworthy since high lactate levels are a hallmark of the Warburg effect in cancer cells (Fan et al., 2025). The relationship between the increased production of lactate and lactylation provides new insights in understanding the association between metabolic and epigenetic changes in cancerous cells. Lactylation is induced owing to high intracellular lactate levels due to Warburg effect as well as due to the re-entry of extracellular lactate via monocarboxylic acid transporters (MCT1/2) or activate G protein-coupled receptors (GPR81) which stimulates the MAPK/ERK pathway further increasing the level of MCT expression as positive feedback regulation. Lactylation promotes tumourigenesis through several mechanisms like stimulating cancer promoting signalling pathways (JAK-STAT and PI3K/Akt/mTOR) which activates certain oncogenes (Sun M. et al., 2025). On the other hand in endometrial cancer, inhibition of histone lactylation correspondingly reduced PI3K/Akt/HIF-1α signalling and lactate generation due to reduction in glycolytic gene (Wei et al., 2024). Also, lactylation in immune cells like tumour-associated macrophages (TAMs) and regulatory T cells (Tregs) induces an immunosuppressive microenvironment which boosts the cancer growth. These cells release inhibitory factors, dampen the responses of natural killer (NK) cells and T cells, facilitate tumor immune evasion, and ultimately support tumour progression (Zhang et al., 2022). Yang et al. (2022) reported significantly higher levels of lactylation in gastric cancer tissues than in adjacent tissues which was associated with poor prognosis (Yang et al., 2022). Hypoxia-induced glycolysis enhances β-catenin stability and expression through increased lactylation, thereby promoting the malignant proliferation of colorectal cancer cells (Miao et al., 2023). Specifically, lactylation at H4K12 has arisen as a potential biomarker for multiple malignancies (Rejili, 2025; Zhang et al., 2025b). Further, lactylation of H3K9 has been reported to activate angiogenesis by increased expression of certain pro-angiogenic genes. Also, DNA repair proteins like MRE11 and NBS1 when lactylated show improved functionality thereby providing resistance to chemotherapy (Sun Y. et al., 2025). Histone lactylation at H3K18 via cyclin B2 (CCNB2), KRT19 and lncRNA NEAT1 were recently reported to promote glycolysis and tumor progression in PDAC, NSCLC and hemangioma (Li et al., 2024; Ma et al., 2025; Zhang et al., 2025a). Furthermore, lactylation of HNRNPA1 promoted metabolic reprogramming via splicing of PKM2 in bladder cancer (Wang T. et al., 2025). Also, histone lactylation promotes the proliferation of BRAF-mutant undifferentiated thyroid cancer cells. *In vitro* studies have demonstrated that BRAFV600E reprograms cellular protein lactylation to enhance anaplastic thyroid cancer (ATC) growth, and that inhibiting the lactylation machinery synergizes with BRAFV600E inhibitors to suppress ATC progression. (Wang et al., 2023). Yang Z. et al. (2023) demonstrated that lactylation enhances the proliferation and metastasis of liver cancer cells by suppressing adenylate kinase 2 (AK2) activity. They performed global lactylome profiling on a prospectively collected hepatitis B virus-related hepatocellular carcinoma (HCC) cohort and identified 9,256 lysine lactylation sites on non-histone proteins. They noticed that lactylation at K28 inhibits the function of AK2, facilitating the proliferation and metastasis of HCC cells (Yang Z. et al., 2023). Based on the ample evidence, lactylation has emerged as an important PTM which links altered carbohydrate metabolism to epigenetic regulation in cancer (Table 1).

**TABLE 1:** Epigenetic effects of various metabolic intermediates in tumourigenesis.

Metabolite	Epigenetic effect	Significance in cancer	References
Glucose	Histone O-GlcNAcylation	Leads to altered chromatic accessibility promoting carcinogen induced hepatocellular carcinoma	Chomphoo et al. (2023)
	Impedes AMPK driven phosphorylation of TET2 at serine99 resulting in destabilization of TET2 causing altered methylation of DNA	Links diabetes to cancer as high glucose provides an environment that can reprogram the epigenome towards an oncogenic state	Wu et al. (2018)
	Downregulation of SIRT1 and increased expression of acetylated high mobility group box 1(HMGB1)	Drives associated signaling pathways leading to glioma progression	Wang Y. et al. (2024)
	PPARɣ and p16 promoter hypermethylation	Promotes carcinogenesis and progression in colorectal and pancreatic cancer	Porcuna et al. (2021), Sun et al. (2023), Xing et al. (2013)
Acetyl CoA	HATs promote global histone acetylation	Modified chromatin structure is observed in tumors	Lee et al. (2013), Miziak et al. (2024)
	Histone acetylation of H3K9 and H3K27	Influences inflammatory, lipogenic, and glycolytic gene promoters involved in complex cancer cell signalling	He et al. (2023)
Lactate	Lactylation of histones; lactylation of H3K9 activates angiogenesis	Stimulates cancer promoting signalling pathways like JAK-STAT and PI3K/Akt/mTOR which activate the oncogenes	Chen et al. (2024) Sun M. et al. (2025)
	Lactylation at H4K12	-Potential biomarker for multiple malignancies	-Rejili (2025), Zhang et al. (2025b)
	Lactylation at H3K18	-Promote glycolysis and tumor progression in PDAC, NSCLC and hemangioma	-Li et al. (2024), Ma et al. (2025), Zhang et al. (2025a)
Fructose	Fructose associated differentially methylated regions (DMRs)	DNA methylation at specific regions overlapping with cell proliferation pathways were observed in colorectal cancers	Devall et al. (2025)
	High fructose derived acetyl CoA increases histone acetylation	Results in upregulation of pro-inflammatory and pro-fibrotic genes in the liver and contributes to carcinogenesis	Assante et al. (2022), Bradshaw (2021)
	Reduced SAM:SAH ratio	reducing DNA and histone methylation. This shift favours expression of lipogenic genes	Gao et al. (2018), Xiao et al. (2022)
Advanced glycation intermediates like MGO, GO	Glycation of histones particularly H3 and altered histone PTM signature leads to disruption of chromatin architecture	Genomic instabilty and cancer progression in breast cancer cell lines, xenografts and patient tumors	Zheng et al. (2019), Knorlein et al. (2023)
	Polysialylation and altered sialyltransferases (STs) expression exerts epigenetic effects	Promotes migration and more aggressive behavior in neuroblastoma and meningoma cells	Scheer et al. (2020), Selke et al. (2021), Knorlein et al. (2023)
	MGO stress via Glyoxylase-1 depletion upregulates DNA methyltransferase 3B (DNMT3B) causing global DNA hypermethylation	Silencing of metastasis-related tumor suppessor genes in triple negative breast cancer cells resulting in enhanced migratory potential	Dube et al. (2023)
	Glycation of Hsp90 chaperone leading to proteasomal degradation of LATS1, a key enzyme of the hippo pathway	Sustained nuclear localization of YAP and TAZ, oncogenic transcriptional co-activators leads to enhanced tumor growth, EMT, and invasion in breast cancer cells	Nokin et al. (2016)
Advanced glycation end products (AGEs)	Alter the epigenome by aberrant epigenetic marks, glycation induced alterations and oncogenetic signalling pathways	Contributes to the epigenetic mechanisms of tumour progression and metastasis in various cancers	Khan et al. (2024)
	AGE-RAGE interaction leads to demethylation of RAGE promoter via upregulation of TET1 leading to stress and activation of downstream signalling in cancer	Leads to stress and activation of downstream signalling however, exact mechanisms remians unexplored and are worth exploring in cancers for improved therapy	Wu et al. (2023)
	Pro-inflammatory pro-oxidant feature of AGEs-RAGE lead to an indelible mark or metabolic imprint in normal breast tissues	“Metabolic memory”created by AGEs seems indispensable for the impending instigation of breast cancer	Palanissami and Paul (2023)
	AGE-RAGE interaction upregulates HMGB1	Promotes tumour growth and suppress antitumor responses in the GL261 and murine glioma	Zhang et al. (2020)
	AGE-RAGE interaction leads to H3K4 hypermethylation and H3K9 hypomethylation of the promoter of NF-κb-p6 gene; induces HATs leading to H3K9 hyperacetylation; Sirtuin1 (SIRT1) downregulation	Contributes to enhanced NF-kappaB signaling leading to inflammation, tumor proliferation; favors enhanced acetylation of STAT3, NF-κb-p65 and FOXO4 leading to establishment of proinflammatory environment	Muthyalaiah et al. (2021)
	AGE-RAGE axis activation leads to induction of expression of DNMT1 mediating hypermethylation of miR-125b and mi199a	Downregulation of miRNAs accelarates the expression of epidermal growth factor receptors (EERBB2/ERBB3) involved in MAPK, PI3K/AKT signalling promoting tumor growth	Muthyalaiah et al. (2021)

### 2.2 High fructose, cancer metabolism and epigenetics

Effects of excess fructose in cancer cells have not been explored adequately, yet emerging evidence indicates that fructose plays a significant role in reshaping cancer metabolism and epigenetic regulation. In certain cancers fructose-derived lipids serve as a preferred carbon source for membrane biosynthesis and growth (Fowle-Grider et al., 2024). Cancer types like HCC, lung cancers and glioblastoma multiforme (GBM) switch to fructose metabolism when glucose is deficient or absent by upregulating GLUT5 expression (Chen et al., 2022; Chen et al., 2025; Santhekadur, 2020). Notably, fructose bypasses the regulatory mechanisms of glycolysis through phosphorylation by ketohexokinase (KHK), yielding fructose-1-phosphate (F1P) enabling cancer cells to sustain bioenergetic and biosynthetic demands independently of glucose availability (Chen et al., 2020; Ting, 2024; Wright et al., 2007).

Dietary fructose is transported through GLUT5 and/or GLUT2 into hepatocytes, independent of insulin (Ferraris et al., 2018). Because fructokinase/KHK is poorly regulated (Tee et al., 2022), fructose metabolism circumvents two rate-limiting steps, regulation by phosphofructokinase and the regulatory influence of insulin (Basciano et al., 2005). Excess fructose is therefore rapidly shunted into *de novo* lipogenesis (DNL) and glycogenesis (Gorczynska-Kosiorz et al., 2024). Sustained fructose overload leads to metabolic dysregulation characterised by insulin resistance and obesity (Basciano et al., 2005; Stanhope et al., 2009). Consequently, elevated acetyl-CoA promotes acetylation of H3K9 and H3K27 at inflammatory, lipogenic, and glycolytic gene promoters (He et al., 2023). Additionally, F1P activates sterol regulatory element binding protein-1 (SREBP-1), a key regulator of lipogenesis, thereby promoting pro-carcinogenic milieu including increased fatty acyl synthase activity, along with promotion of migration and invasion via mTOR pathway (Brouwers, 2022; Hannou et al., 2018; Kim et al., 2012). Moreover, ALDOB (aldolse B) which is critical for fructose metabolism has also been reported to promote fructose dependent liver cancer cell metastasis (Bu et al., 2018).

#### 2.2.1 Epigenetic consequences of fructose induced metabolic shifts and tumourigenesis

Fructose’s influence extends beyond metabolic rewiring to directly affect epigenetic landscape. Excess fructose been linked to the risk of pancreatic, prostate and small intestinal cancers (Aune et al., 2012; Carreno et al., 2021; Goncalves et al., 2019; Port et al., 2012; Senavirathna et al., 2023).

A distinct feature of fructose metabolism is its preferential routing through the non-oxidative branch of Pentose Phosphate Pathway (PPP). Increased activity of glucose-6-phosphate dehydrogenase and transketolase in cancer cells enhances ribose-5-phosphate availability for nucleotide and uric acid synthesis thereby providing proliferative advantages (Benito et al., 2017). Elevated fructose flux also overloads fructose-6-phosphate and glyceraldehyde-3-phosphate which are shunted for production of acetyl-CoA (Cho et al., 2018), which leads to increased histone acetylation thereby leading to upregulation of pro-inflammatory and pro-fibrotic genes contributing to hepatic tumourigenesis (Assante et al., 2022; Bradshaw, 2021). Hence, increased fructose intake can drive diverse epigenetic modifications including altered DNA methylation, histone marks and non-coding RNA expression (DiStefano, 2020; Sud et al., 2017).

#### 2.2.2 Fructose driven epigenetic crosstalk and metabolic dysregulation

In cancers lacking KHK expression, fructose metabolism can be outsourced to the liver where tumour cells hijack liver-derived lipids especially lysophosphatidylcholines (LPCs) to support accelerated proliferation (Park et al., 2021). Such metabolic cross talk has been observed in animal models of melanoma, breast cancer, and cervical cancer and HCC wherein high fructose consumption increased tumour burden without impacting the body weight or insulin levels (Fowle-Grider et al., 2024). Beyond lipogenesis, high fructose intake enables rapid production of dicarbonyl precursors, at even higher rates than glucose (Gensberger et al., 2013; Hattori et al., 2021). This in turn promotes AGEs accumulation, contributing to peripheral insulin resistance, dyslipidemia, inflammation, elevated uric acid levels all of which are tightly linked to tumourigenesis (Collino, 2011).

Population studies have reinforced the above mechanistic observations. A large cohort-based study involving 435,674 individuals found that dietary fructose specifically increased the risk of intestinal cancers whereas other added sugars (including mono-and disaccharides) were associated with esophageal adenocarcinoma (Tasevska et al., 2012). In colorectal cancers among African-American individuals, excess fructose intake corresponded to unique differential methylated regions (DMR) in DNA affecting flux through pathways such as fatty acid metabolism and glycolysis. These modifications also alter LDHA expression, highlighting an interaction between diet, metabolic reprogramming, and racial disparities in cancer susceptibility (Devall et al., 2025).

Excess fructose consumption is a major contributor to NAFLD and non-alcoholic steatohepatitis (NASH) which often progresses to hepatic carcinoma. DNL driven lipid accumulation, inflammation and oxidative stress create a pro-oncogenic hepatic environment (Dewdney et al., 2020; Geidl-Flueck and Gerber, 2023). Fructose metabolism additionally depletes S-adenosylmethionine (SAM), thereby reducing the SAM/S-adenosylhomocystein (SAH) ratio thereby reducing DNA and histone methylation. This shift favours expression of lipogenic genes and exacerbates fatty liver progression. (Kim and Min, 2020; Verma and Lindroth, 2025). Because the SAM:SAH ratio (methylation index) dictates methylation potential, its reduction leads to widespread hypomethylation (Gao et al., 2018; Xiao et al., 2022). Thus, from acetylation to methylation, fructose driven multiple mechanisms contribute to altered epigenetic changes driving cancer metabolism (Table 1).

## 3 Glycation intermediates, chromatin glycation, and epigenetics in cancer

Glycolysis and other connected pathways such as gluconeogenesis and PPP are known to spontaneously produce highly reactive α-ketoaldehydes (dicarbonyl compounds) as by-products namely, glyoxal (GO), methylglyoxal (MGO) and 3-deoxyglucosone (3-DG). Since cancer cells hyperactively utilize glucose, it is pertinent that such cells would produce higher levels of these dicarbonyl compounds also (Moldogazieva et al., 2019). These three metabolites are potent glycation agents and therefore these are collectively known as AGIs which cause the formation of AGEs. Three carbon sugars, primarily Glyceraldehyde-3-phosphate (G3P) and Dihydroxyacetone phosphate (DHAP) are known to produce minute amounts of MGO which in turn reacts with DNA and proteins in a non-enzymatic manner under physiological conditions (Phillips and Thornalley, 1993). Mammalian cells have enzymatic mechanism, namely, glyoxylases to defend against the MGO but immunohistochemistry analyses revealed high levels of MGO and low glyoxylase activity in 102 colorectal cancer patients with advanced stage tumours compared to early-stage tumours suggesting a pro-tumour role of MGO (Chiavarina et al., 2017). Apart from spontaneous production of MGOs, enhanced utilisation of sugars promotes ROS generation due to redox imbalance in cancer cells (Nakamura and Takada, 2021). This in turn promotes both sugar oxidation and lipid peroxidation. Peroxidation of lipids especially of polyunsaturated fatty acids (PUFA) by ROS promotes the formation of reactive carbonyl species (RCS) namely, malondialdehyde (MDA) and 4-hydroxynonenal (4-HNE) both of which further promote AGEs production (Barrera et al., 2018; Li et al., 2022). Glutathione (GSH) plays a vital role in mitigating oxidative damage and detoxification of MGO via the glyoxylase system. Oxidative stress depletes reduced GSH, thereby helping in MGO build-up which in turn promotes accumulation of AGEs (Reddy, 2023). AGEs further promote ROS build up by activating receptors of AGEs therefore making the whole scenario cyclic and never ending in cancer cells.

Non-enzymatic glycation on DNA and histones affects the histone-histone and histone-DNA interactions in chromatin which in turn influence epigenetic regulation. Basic nature and long half-lives of histone proteins make them highly susceptible to glycation and the emerging evidence indicates that glycation-mediated epigenetic alterations can influence cellular function by modulating the expression of key metabolic genes. Sugars, and their derivatives including MGO, glyoxal and dicarbonyls are highly reactive glycation intermediates, where the extent of glycation reactions depends on the time and concentration of AGIs (Rehman et al., 2022; Tessier, 2010).

### 3.1 AGIs and histone modification

Histone modification is known to change the gene expression dynamics. Cellular metabolism is closely associated with methylation and acetylation of histones (Fan et al., 2015). Specific histone tail PTMs promote either heterochromatin or euchromatin formation at precise locations in the genome (Jenuwein and Allis, 2001). Such modifications are known to play a pivotal role in tumourigenesis (Thompson et al., 2013). Histone tails having lysine and arginine are predominantly vulnerable to modification by glyoxal and MGO (Ray et al., 2022). MGO has been shown to react more with arginine compared to lysine on histone tails. N terminus of H3/H2A/H2B/H4 was found to be extensively modified (H3 being more susceptible) due to MGO which in turn affected canonical PTMs such as acetylation on these histone tails in multiple cancer cell lines (Galligan et al., 2018; Zheng et al., 2019).

Enzymes involved in the PTMs of histones use a wide variety of carbohydrate derived metabolites that serve as precursors. Examples include acetyl-CoA diverted towards histone acetyltransferases (HATs) wherein increased acetylation opens the chromatin structure making it more prone to aberrant expression stimulating tumourigenesis (Bradshaw, 2021; Fan et al., 2015). Similarly, phosphorylation of histone H3 is catalysed by the glycolytic enzyme PKM2, leading to removal of histone deacetylase HDAC3 from chromatin, resulting in increased H3K9 acetylation thereby activating oncogene expression promoting tumourigenesis (Yang et al., 2012). Another important post-translational modification is Histone GlcNAcylation (O-GlcNAcylation) wherein sugars like glucose, fructose, or glycolysis by-products glycosylate histones H3 and H4 disrupting the nucleosome assembly, reducing histone acetylation and reducing the activity of histone demethylases like EZH2 leading to dysregulated gene expression and increased cancer progression (Khan et al., 2024; Yang Y. H. et al., 2023; Zheng et al., 2019).

MGO adducts on histone tails neutralise the positive charge of histones affecting the tight binding of histone proteins with DNA thereby promoting decompaction of chromatin (Zheng et al., 2019). The consequences of MGO levels on chromatin are dose-dependent: low-level MGO neutralization leads to loosening of chromatin and transcriptional upregulation (resembling acetylation), whereas chronic/high MGO exposure causes crosslinks among histones and DNA, inducing chromatin compaction and gene silencing. Glycation of histones due to MGO in breast cancer cell lines, xenografts and patient tumours results in disruption of chromatin structure and function contributing to genomic instability and cancer progression (Knorlein et al., 2023; Zheng et al., 2019). MGO induced histone glycation “rewrites” the epigenetic histone code by adding bulky, irreversible marks, and this remodelling of chromatin structure can profoundly influence gene expression (Knorlein et al., 2023; Scumaci and Zheng, 2023). Glycation due to MGO and GO also shows epigenetic effects on cancer development by interfering with the expression of sialyltransferases (STs) (Scheer et al., 2020; Selke et al., 2021; Schildhauer et al., 2023b). These STs play an important role in cell migration, invasion, and promoting stemness (Falconer et al., 2012; Sato and Kitajima, 2021). Polysialylation has been reported on neural cell adhesion molecules (NCAM) in neuroblastoma cells (Gluer et al., 1998; Schildhauer et al., 2023b) analyzed the effect of glycation on mRNA levels of STs and they observed the upregulation of STs mRNA levels in LN22 cells (Schildhauer et al., 2023b). A possible mechanism that can account for the association of glycation with expression of STs is by interaction with the histones. Emerging evidence suggests that the glycation of histones at arginine and lysine residues may epigenetically modulate the DNA thereby, affecting the transcriptional changes associated with aberrant sialylation and STs expression (Knorlein et al., 2023). Therefore, polysialylation due to increased glycative stress in cancer, may contribute to enhanced invasion and a markedly more aggressive phenotype (Table 1). Thus, MGO mediated modifications exert a widespread effect on histone PTMs thereby, thereby influencing the nucleosome assembly, stability, and overall chromatin organization. Apart from MGO induced histone modifications, ribose and its derivatives such as the ubiquitous ADP-ribose, have also been noticed to glycate all four core histones as well as the linker histone H1. In solution, the core histones react more rapidly with ribose than H1, however, within chromatin, the pattern reverses and ribose modifies the linker histone more quickly than the core histones (Maksimovic and David, 2021). Maksimovic et al. (2020) demonstrated that similar to MGO glycation, ribose glycation alters the histone properties and is involved in epigenetic changes. To confirmed this, they cultured 293T cells with increasing concentrations of D-ribose and studied the PTMs on histones by Western blotting. They observed that the signals for general acetyl-lysine (pan-KAc) and histone H3 lysine-9 dimethylation (H3K9Me2) were reduced upon ribose treatment, whereas the signal for H3 lysine-36 trimethylation (H3K36Me3) remained unchanged (Maksimovic et al., 2020).

### 3.2 AGIs and DNA methylation

Altered DNA methylation, a classic marker of epigenetic regulation, is directly influenced by glycolytic intermediates and any perturbation in this process can significantly contribute to tumourigenesis (Maleknia et al., 2023). Aberrant DNA methylation mediated by methyl transferase can promote tumourigenesis through multiple mechanisms. Hypermethylation of promoter regions can lead to silencing of tumour suppressor genes, such as Fructose-1,6-bisphosphatase (FBP-1) and Fructose-2,6-bisphosphatase (FBP-2), which are required to stimulate gluconeogenesis and inhibit glycolysis. This silencing supports predominantly glycolytic phenotype that promotes tumour cell growth (Wang M. et al., 2025). Conversely, hypomethylation of promoter regions can lead to inappropriate activation of oncogenes or genes promoting glucose uptake, such as Glucose Transporter 1 (GLUT1) and HK2, enhancing aerobic glycolysis and overall tumourigenesis (Goel et al., 2003; Wolf et al., 2011).

MGO has been shown to promote metastasis in breast cancer via activation of the MEK/ERK/SMAD1 pathway (Nokin et al., 2019). Similarly, breast cancer models have demonstrated that MGO induced stress achieved through Glyoxylase-1 (GLO1) knockdown dramatically upregulates DNA Methyltransferase 3 beta (DNMT3B) and causes global DNA hypermethylation of metastasis-suppressor genes thereby promoting metastatic progression. Collectively these findings establish MGO as an oncometabolite downstream of the Warburg effect (Dube et al., 2023). Notably, MGO scavengers such as carnosine and aminoguanidine mimic DNMT inhibitors by down-regulating DNMT3B, reverse hypermethylation and re-activating silenced genes, ultimately suppressing metastasis (Nokin et al., 2016) (Table 1).

## 4 AGE-RAGE axis in cancer

Cancerous cells continuously produce high levels of AGEs due to aberrant metabolic activity characterised by enhanced aerobic glycolysis and increased generation of glycolytic intermediates (Leone et al., 2021; Vaupel and Multhoff, 2021). PTMs induced by AGEs and their intermediates alter the protein structures and functions leading to glycative stress, stimulation of inflammation and increased oxidative stress. These processes contribute to DNA modification and dysregulated cell signalling pathways that promote cancer cell proliferation and survival (Alfarouk et al., 2021; Chiavarina et al., 2017).

AGEs exhibit their deleterious effects primarily through interaction with certain receptors which may exist in soluble form or be expressed at cell surface. Key AGEs binding proteins include AGE-R1 (oligosaccharyltransferase-48), AGE-R2 (80KD-phosphoprotein), AGE-R3 (galectin-3) and RAGE (Bierhaus et al., 2005; Yan et al., 2010). AGEs largely display their effects by interacting with cell surface receptor RAGE, which is the best characterised receptor for AGEs. RAGE is an approximately 45-kDa protein, multiligand receptor belonging to the immunoglobulin superfamily and is expressed on the cell-surface of endothelium, vascular smooth muscle cells, and invading mononuclear phagocytes (Cross et al., 2024; Schmidt et al., 2001). Its widespread tissue distribution underscores its pivotal role in governing cellular properties through various pathways/mechanisms.

Enhanced AGEs formation induces RAGE expression associated with many chronic conditions such as diabetes, renal dysfunctions, cardiovascular diseases, and aging. Although, the involvement of the AGE/RAGE is well established in these disorders, its role in cancer development remains unexplored. The activation of RAGE by AGEs in cancer cells triggers a cascade of intracellular signalling that ultimately leads to the upregulation of glycolytic enzymes, promoting glycolysis and Warburg effect (Zhou et al., 2022). The AGE-RAGE interaction also activates the STAT3/Pim1/NFAT axis, which maintains a high transcription rate of RAGE through a positive feedback mechanism further amplifying RAGE expression (Meloche et al., 2011; Zhang et al., 2020) reported elevated expression of RAGE by AGEs can promote tumour growth and suppress antitumour responses in the GL261 glioma model through upregulation of HMGB1. Additionally, increased HMGB1 released within the tumour microenvironment further enhances RAGE expression, increased tumour inflammation and angiogenesis in GL261 cell line and murine gliomas (Chen et al., 2014; Zhang et al., 2020).

Glycative stress resulting from AGE accumulation and AGE-RAGE axis also activates crosstalk between the tumour cells and its microenvironment to generate hypoxia, mitochondrial dysfunction, epigenetic modifications further enhancing the tumourigenesis and cancer progression (Muthyalaiah et al., 2021; Neviere et al., 2016; Zhang et al., 2024). AGE-RAGE interaction at the cell-surface activates the cascade of cell-signalling which results in moderation of gene expression affecting the cell functioning. AGE-RAGE ligation enhances cytosolic and mitochondrial reactive oxygen species generation, cytokine production, adhesion molecule expression, endothelial-1, plasmin activator inhibitor 1 production, release of growth factors (such as TNF-α), recruitment of inflammatory cells, oxidative stress, stimulation of pro-inflammatory and pro-coagulant pathways along with smooth muscle cells and fibroblast proliferation. (Bierhaus et al., 2005; Bierhaus et al., 2001; Goldin et al., 2006). Molecular mechanism and signalling pathways linking the AGE-RAGE axis with tumourigenesis includes activation of NOX-2, NF-κB, SP-1, expression of MMP1, 2, 3, 7, 9 and 10; B-cell lymphoma-extra-large (Bcl-xL), phosphorylation of AKT and mTOR (a serine/threonine protein kinase in the PI3K-related kinase family, responsible for cancer cell proliferation and pro-survival signals), phosphorylation of ERK 1/2, p38 MAPK, STAT-3 and 70-kDa ribosomal protein S6 kinase (p70S6K1), phosphorylation of GSK-3β (responsible for ECM remodelling and hence metastasis), rise in cytokines IL-1β and IL-8 (responsible for inflammation; and metastasis) and down regulation of NRF-2, Bcl-2 (anti-apoptotic protein), p53 (tumour suppressor) (Bansal et al., 2012; Geicu et al., 2020; Korwar et al., 2012; Palanissami and Paul, 2023; Sorci et al., 2013; Sparvero et al., 2009). Despite the insights, the precise molecular mechanisms by which the above signalling aberrations drive cancer development and progression remain improperly understood.

Waghela et al., 2021 investigated multiple molecular pathways associated with the process of apoptosis, autophagy, and necrosis that are influenced by the AGE-RAGE signalling in cancerous cells. Their findings demonstrated that the AGE-RAGE interaction perturbs the cellular redox balance and modulates the cell death signalling in cancerous cells during the malignant progression (Waghela et al., 2021). More recently, Xia et al., 2024 identified the differential expression of 292 genes in gastric cancer and 541 in T2DM with 104 genes shared between two conditions. A crosstalk mechanism has been proposed for the overlapping genes’ differential expression in cancer and diabetes due to convergent AGE-RAGE signalling pathways that induce hypoxia, autophagy, epigenetic modification and mitochondrial dysfunction. (Muthyalaiah et al., 2021; Xia et al., 2024). Furthermore, enhanced expression of RAGE due to AGEs accumulation has been linked to multiple cancer types, by establishment and maintenance of a chronic inflammatory state (Ishiguro et al., 2005; Kuniyasu et al., 2002). Chen et al. (2014) demonstrated that genetic depletion of RAGE in the tumour microenvironment prolonged survival in glioma bearing mice by reducing the tumour associated inflammation and suppressing angiogenesis (Chen et al., 2014). Importance of AGE-RAGE interaction in prostate cancer development was further confirmed by incubation of DU145 cells with AGEs resulting in increased invasive potential and growth of these cells (Ishiguro et al., 2005; Khoo et.al., 2023). Therefore, metabolic re-programming inside the cancerous cells is effective enough to support the AGE-RAGE signalling cascade generating malignant features of cancer through increased cell proliferation, survival, tumour promoting inflammation, apoptotic evasion, increased oxidative stress, genomic instability, angiogenesis and immune suppression leading to the TME and metastasis.

Association of AGE-RAGE axis with epigenetic changes in cancer has also been explored via different mechanisms such as upregulation of the expression of TET1 which is involved in DNA demethylation and the induction of stress and activation of downstream signalling pathways (Turner, 2017; Wu et al., 2023). Wu et al., have comprehensively reviewed the epigenetic impact of AGEs on RAGE gene expression. They observed that the treatment of liver cells with CML and CEL (carboxyethyl lysine) regulates the RAGE expression epigenetically by promoting the demethylation of the RAGE promoter region through elevated levels of TET1. Epigenetic impact of AGEs on RAGE gene expression has also been implicated in other chronic diseases including diabetes and diabetic retinopathy where hypomethylation of RAGE gene promoter results in increased levels of IL-1β, IL-6 and TNF-α in PBMC of patients (Kan et al., 2018; Kang and Yang, 2020). Hence, AGEs can also modulate RAGE gene expression through epigenetic modifications in cancer cells (Table 1); however, the precise mechanisms underlying this epigenetic regulation remain to be elucidated.

## 5 Extracellular matrix perturbations due to epigenetic effects of glycation

Glycation of ECM proteins alter ECM properties and promote cancer cell invasion and metastasis. During neoplastic progression ECM becomes highly disorganized facilitating cancer cell transformation and invasion. (Palanissami and Paul, 2023). AGEs preferentially accumulate in long-lived, low turnover proteins, a characteristic shared by many ECM components during molecular ageing (Zgutka et al., 2023). AGE modified proteins are recognized by AGE binding proteins, thus glycated ECM components are capable of triggering RAGE-dependent metastatic neoplastic progression (Rojas et al., 2018). The pro-inflammatory environment generated by AGE mediated RAGE activation further facilitates tumour growth and progression by promoting ECM remodelling, angiogenesis and metastasis (Riehl et al., 2009).

Additional studies have shown that glycation by MGO can alter ECM components like collagen, fibronectin and laminin promoting cancer progression and invasiveness (Leone et al., 2021). Schildhauer et al. (2023b) reported that glycation differentially affects ECM components involved in glioblastoma invasion, upregulating TGFβ, brevican and tenascin C in LN229 and U251 cell lines. Exposure to physiological concentrations of MGO enhanced aggressiveness of glioblastoma cells whereas higher concentrations were cytotoxic (Schildhauer et al., 2023a).

Accumulation of AGEs within the ECM also increase matrix stiffness, actively playing a role in tumour progression and altering treatment response. Jang et al. (2022) demonstrated that AGEs accrued in the ECM altered the fibroblast phenotype within a three-dimensional collagen matrix, through upregulation of AGE-RAGE interaction and integrin mediated mechanotransduction, promoting fibroblast activation towards a cancer associated fibroblast (CAF) like phenotype. These effects were attenuated by neutralizing antibodies against RAGE or by inhibition of focal adhesion signalling. Importantly, reducing matrix stiffness diminished fibroblast activation, highlighting ECM associated AGEs as potential targets for modulating tumour stroma and metastatic progression (Jang et al., 2022). Long term persistence of AGEs and sustained activation of RAGE generate a chronic proinflammatory and pro-oxidant environment that leaves an “indelible metabolic imprint” on tissues, as observed in normal breast tissues. Consequently, the AGE/RAGE complex has the capacity to drive pre-neoplastic transformation, ultimately contributing to cancer initiation and progression through permanent biological changes, mediated by ECM alterations (Palanissami and Paul, 2023).

## 6 Conclusion

Metabolic reprogramming and epigenetic remodelling are fundamental mechanisms that enable cancer cells to sustain growth, survival, and metastatic progression. Reducing sugars and their reactive metabolites serve as critical mediators linking altered cancer metabolism to epigenetic dysregulation. Increased glycolytic flux elevates intracellular levels of glucose, fructose, lactate, glyoxal, methylglyoxal, and 3-deoxyglucosone, which induce DNA hypomethylation and histone modifications such as hyperacetylation, glycation, and lactylation. These alterations promote oncogenic transcriptional programs while down-regulating tumour-suppressor signalling, thereby facilitating tumour progression. High glucose availability also enhances histone O-GlcNAcylation and disrupts AMPK and TET activity, altering chromatin accessibility. Concurrently, activation of the polyol pathway redirects glucose metabolism toward fructose synthesis, increasing oxidative stress, inflammation, and chemoresistance while maintaining NF-κB signalling and oxidative stress. Elevated glucose further modulates SIRT1 activity, promoting chromatin relaxation, proliferation, and invasion. Excess glucose and fructose accelerate AGE formation and activate the AGE-RAGE axis, triggering oxidative stress, inflammatory signalling, mitochondrial dysfunction, and aberrant gene regulation. Downstream activation of NF-κB, MAPK, PI3K/AKT/mTOR, and STAT3 pathways enhances proliferation, angiogenesis, metastasis, and apoptosis resistance, while persistent RAGE activation maintains a chronic pro-inflammatory tumour microenvironment. This reinforces the Warburg phenotype, stromal remodelling, immune evasion, and therapeutic resistance. Glycation-dependent histone modifications may further upregulate sialyltransferases, promoting polysialylation, migration, and stemness. AGE accumulation within the extracellular matrix stiffens the stroma and fosters a metastatic niche, while methylglyoxal-mediated protein modification intensifies invasion.

In conclusion reducing sugars and their active metabolites do not act merely as metabolic substrates but as potent modulators of the cancer epigenome and microenvironment. Understanding the metabolic and epigenetic interplay through glucose and other glycolytic intermediates provides novel insights into cancer etiology and identifies potential therapeutic targets, including modulation of sugar metabolism, inhibition of the AGE-RAGE axis, and restoration of epigenetic homeostasis. Understanding the mechanistic interplay between glycation, histone PTMs, and chromatin remodeling not only deepens our insight into metabolic-epigenetic crosstalk but also highlights potential therapeutic opportunities such as MGO scavengers or epigenetic modulators to counteract the oncogenic effects of glycative stress. Future research should focus on delineating these mechanistic interconnections in different tumour types, identifying metabolic biomarkers that predict cancer susceptibility, progression, and response to treatment. Such insights will be instrumental in developing integrative strategies that combine metabolic control with epigenetic understanding to combat cancers more effectively.
